# Supplementary material for: Regulation of symbiotic interactions and primitive lichen differentiation by UMP1 MAP kinase in Umbilicaria muhlenbergii
Source: Nat Commun. 2023 Nov 1;14:6972. doi: 10.1038/s41467-023-42675-8 (PMC10620189; doi:10.1038/s41467-023-42675-8)
Supplement: Supplementary file 1 — Supplementary information [file 41467_2023_42675_MOESM1_ESM.pdf]

Regulation of symbiotic interactions and primitive lichen differentiation by *UMP1* MAP kinase in *Umbilicaria muhlenbergii*

Yanyan Wang[1,2], Rong Li[1], Diwen Wang[2], Ben Qian[1], Zhuyun Bian[2], Jiangchun Wei[1], Xinli Wei[1*], and Jin-Rong Xu[2*]

[1] State Key Laboratory of Mycology, Institute of Microbiology, Chinese Academy of Sciences, Beijing, 100101. China.

[2] Dept. of Botany and Plant Pathology, Purdue University, West Lafayette, IN 47907. USA.

TABLE OF CONTENTS:

Supplementary Figures

Supplementary Figure 1. Formation of the fungal-algal complex on glass.

Supplementary Figure 2. Algal or fungal cells cultured separately on cellulose membranes for 3-month.

Supplementary Figure 3. Effects of MEK inhibitors on fungal-algal interactions.

Supplementary Figure 4. Phylogenetic analysis with fungal MAPKs.

Supplementary Figure 5. Sequence alignment of the subdomain VIII region with the TEY dual phosphorylation site.

Supplementary Figure 6. The *UMP1* gene replacement mutant and complementation.

Supplementary Figure 7. Assays for the expression of Ump1-GFP fusion protein.

Supplementary Tables

Supplementary Table 1. Number of algal cells with FDA and chlorophyll fluorescence signal in primary lichen thalli and three-month-old algal samples.

Supplementary Table 2. Primers used in this study.

Supplementary Table 3. Accession numbers and genome locations of MAPKs used in phylogenetic analysis.

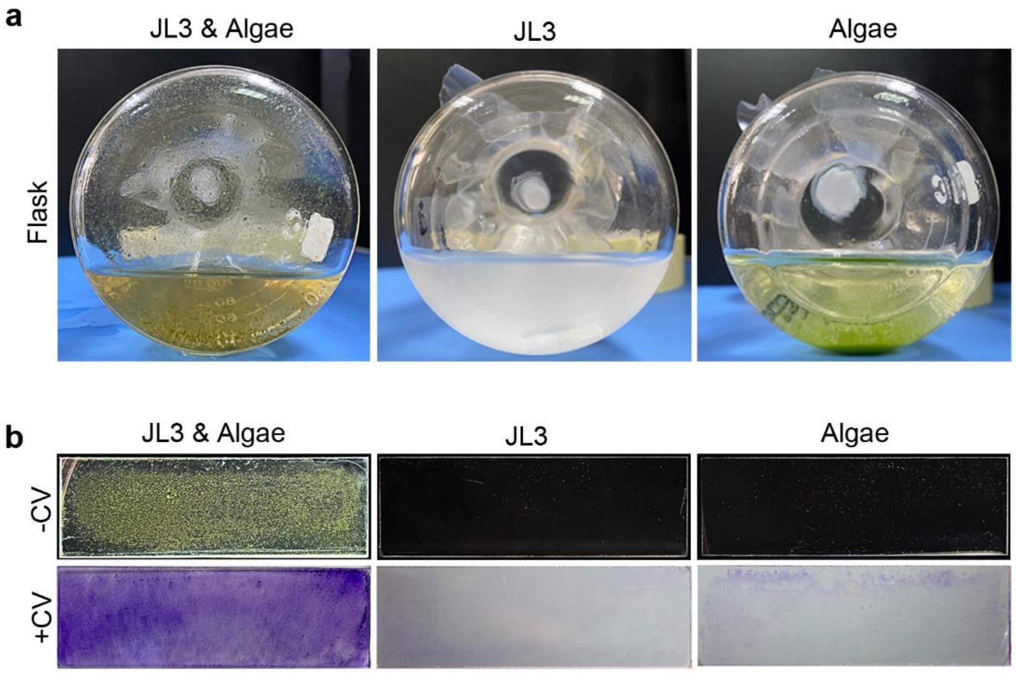

Supplementary Figure 1. Formation of the fungal-algal complex on glass.

a. Flasks containing marked 10-day-old 0.1×PDB cultures were examined for cell masses adhered to the bottom after vigorous shaking. A granular layer of cell masses adhered to the bottom of flasks was observed only in co-cultures of *U. muhlenbergii* JL3 and *T. jamesii* algal cells (1:10). **b**. Slide glasses submerged in the marked 0.1×PDB cultures for 10 days were gently rinsed and directly examined or examined after staining with crystal violet (CV). A greenish (no staining) or purplish (after CV staining) layer was observed in fungal-algal cocultures but not in JL3 and *T. jamesii* cells cultured alone. Observations of the fungal-algal complex on glass came from five independent experiments(*n=5*) with no significant differences between replicates.

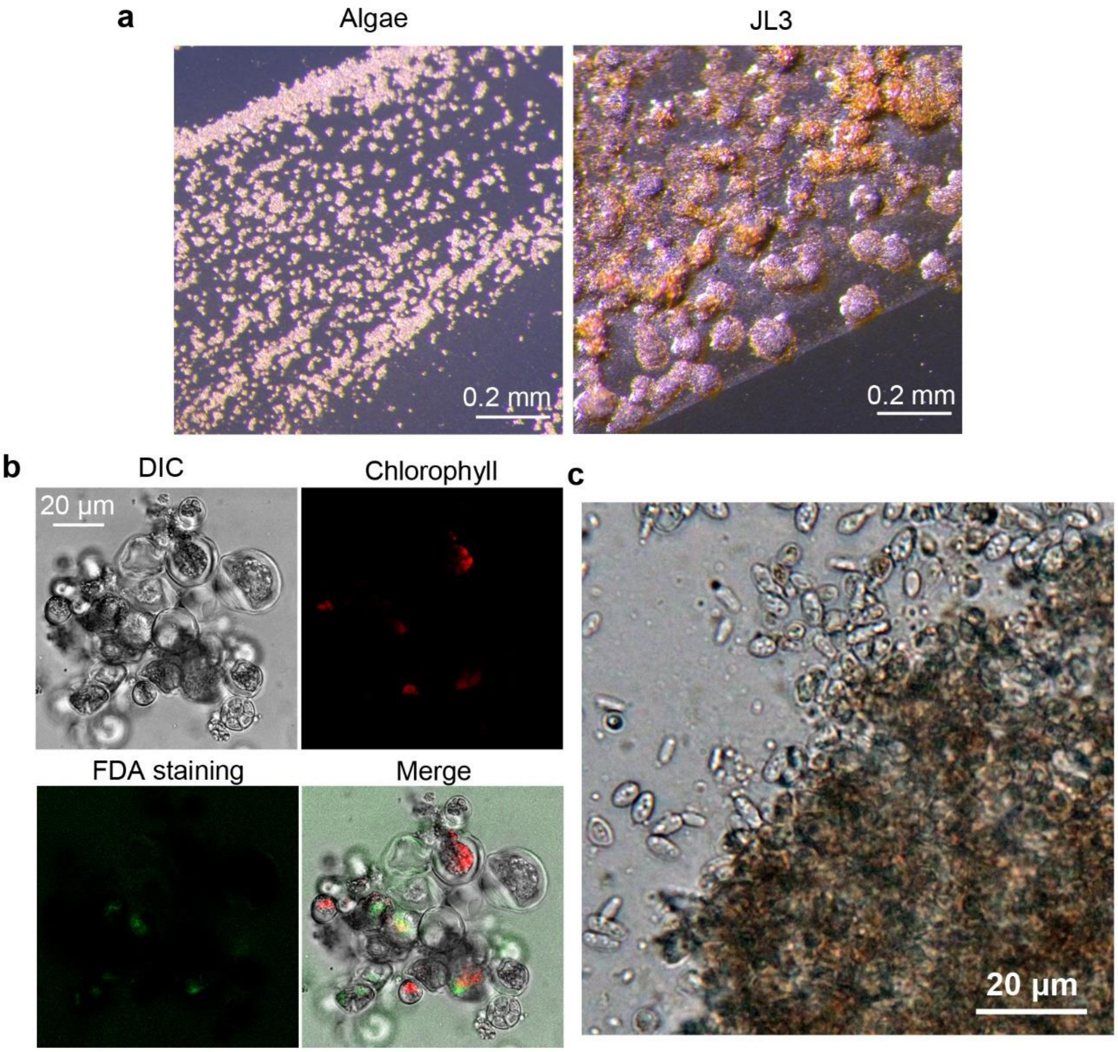

Supplementary Figure 2. Algal or fungal cells cultured separately on cellulose membranes for 3-month.

a. Algal or fungal cells (JL3) cultured on cellulose membranes for three months. Similar results in three independent experiments (*n=3*), the most representative results were presented. **b.** Algal cells were stained with FDA and observed by DIC and epifluorescence microscopy. 92.3 ± 0.9% of the algal cells appeared to be dead or damaged and lack chlorophyll autofluorescence. The statistics were presented in Supplementary Table 1. **c.** Yeast cells of *U. muhlenbergii*. The aggregated cells tend to be brownish and some of yeast cells were empty or dead.

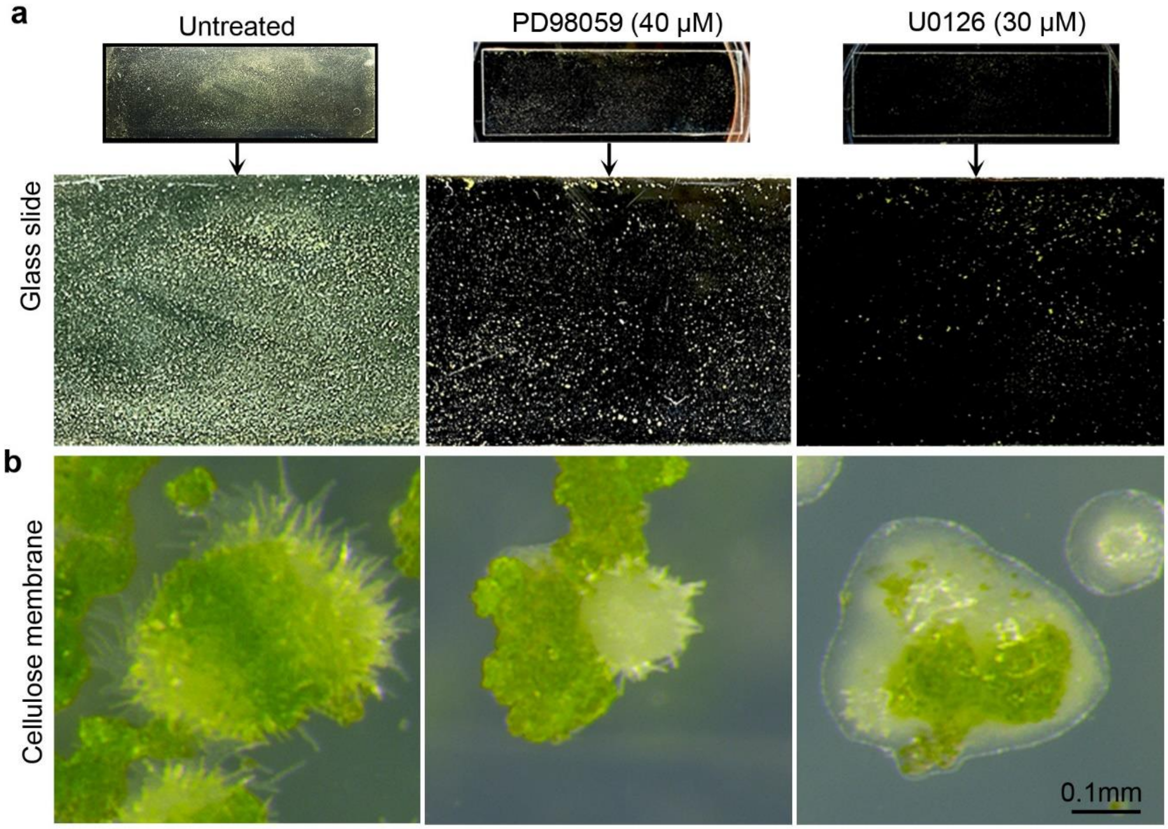

Supplementary Figure 3. Effects of MEK inhibitors on fungal-algal interactions.

a. Slide glasses submerged in the 0.1×PDB cultures of fungal and algal cells (1:10) with or without labelled MEK inhibitors for 10 days were gently rinsed before examination. U0126 was more effective than PD98059 in inhibiting the formation of greenish fungal-algal symbiotic complex. **b.** The mixture of fungal-algal cells (1:10) was cultured on cellulose membranes laid over 0.1×PDA with or without labelled MEK inhibitors for 10 days. Treatments with both inhibitors, particularly U0126 significantly reduced the formation of fungal-algal symbiotic complexes, resulting the growth of whitish yeast cells and greenish algal cells separately. U0126 inhibited pseudohyphal growth and eliminated hairy appearance of the fungal-cell masses. Plates from three independent experiments (*n=3*) were examined and all had similar results.

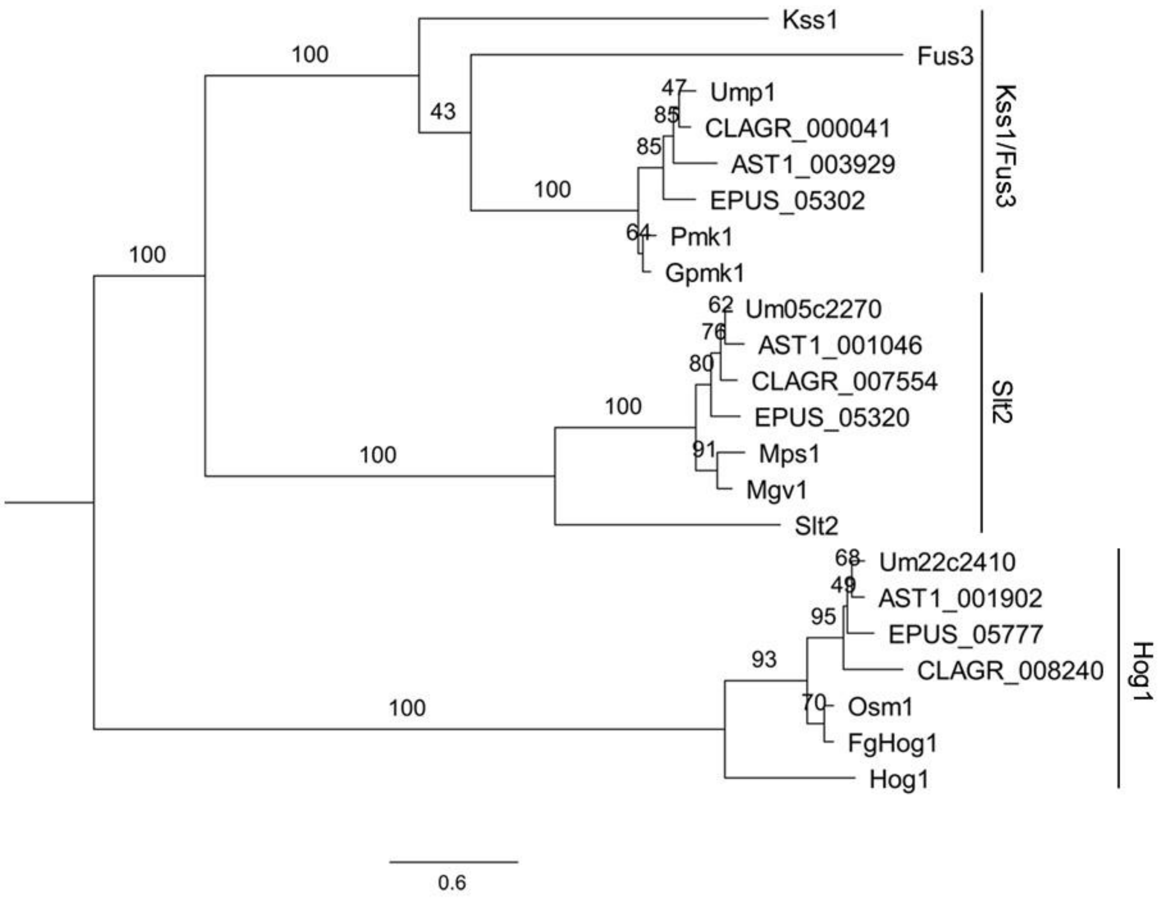

Supplementary Figure 4. Phylogenetic analysis with fungal MAPKs.

A maximum likelihood phylogenetic tree of MAPKs from *Saccharomyces cerevisiae* (Kss1, Fus3, Slt2, Hog1), *Magnaporthe oryzae* (Pmk1, Mps1, Osm1), *Fusarium graminearum* (Gpmk1, Mgv1, and FgHog1), *Umbilicaria muhlenbergii* (Um), *Cladonia grayi* (CLAGR), *Endocarpon pusillum* (EPUS), and *Acarospora strigata* (AST). Selected protein accession numbers are listed in Supplementary Table 3. Bar, 0.6 substitution per site. Number on the branch represent the bootstrap support values.

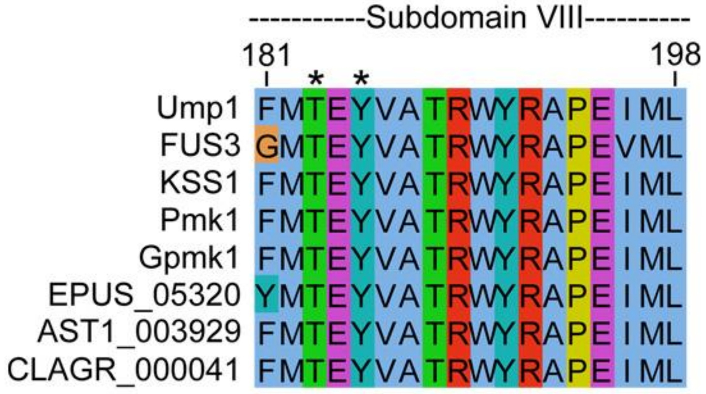

Supplementary Figure 5. Sequence alignment of the subdomain VIII region with the TEY dual phosphorylation site.

Amino acid sequences of Ump1 (181-198 aa) and corresponding regions of its orthologs from *Saccharomyces cerevisiae* (Fus3, Kss1), *Magnaporthe oryzae* (Pmk1), *Fusarium graminearum* (Gpmk1), *Cladonia grayi* (CLAGR), *Endocarpon pusillum* (EPUS), and *Acarospora strigata* (AST) are aligned with ClustalW. The TEY (Thr-Glu-Tyr) dual phosphorylation site in subdomain VIII are marked with * on the top.

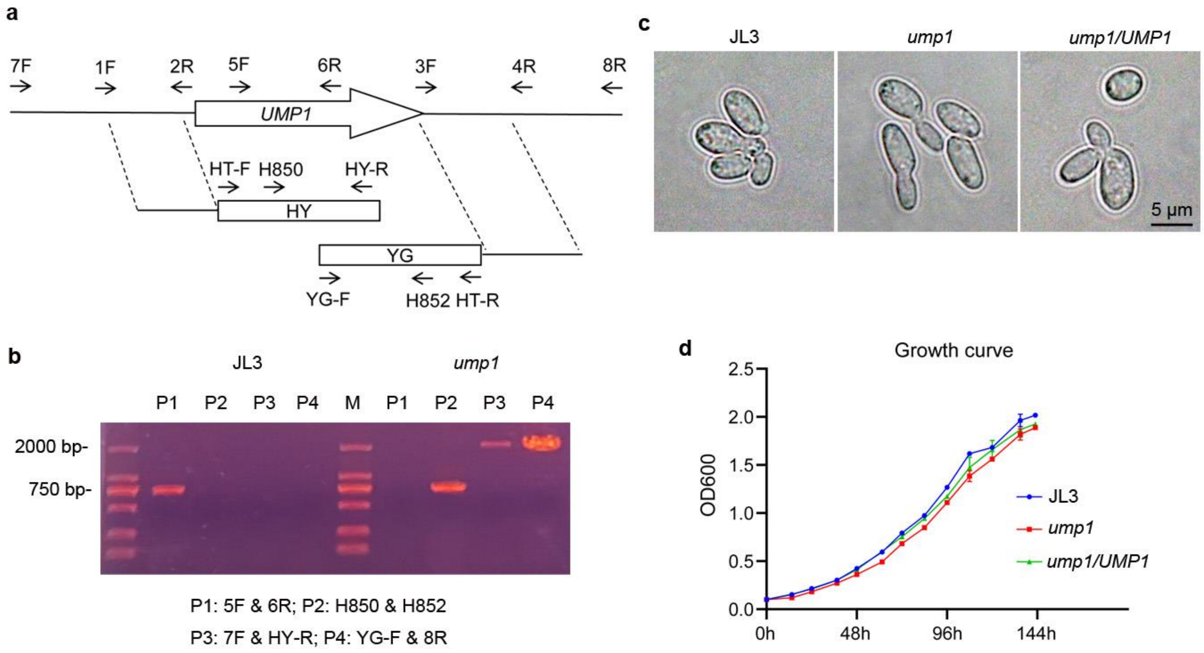

Supplementary Figure 6. The *UMP1* gene replacement mutant and complementation.

a. Diagram of the *UMP1* gene, fragments of the *hph* cassette (HY and YG), and PCR primers used to generate and verify the *ump1* deletion mutant. **b.** Verification of the *UMP1* gene replacement event by PCR with marked primers in the *ump1* mutant ud1. JL3 is the wild-type strain. **c.** Yeast cells of JL3, *ump1* mutant, and *ump1/UMP1* complementation transformant. **d.** Growth rate of the same set of strain was measured with OD_{600}. Yeast cells of each strain were inoculated into 200 ml PDB, adjusted to OD_{600} to 0.1, incubated at 25ºC with gentle shaking (100 rpm), and measured every 12 h for 144 h. Means and standard deviations were calculated from data from three (*n=3*) independent replicates.

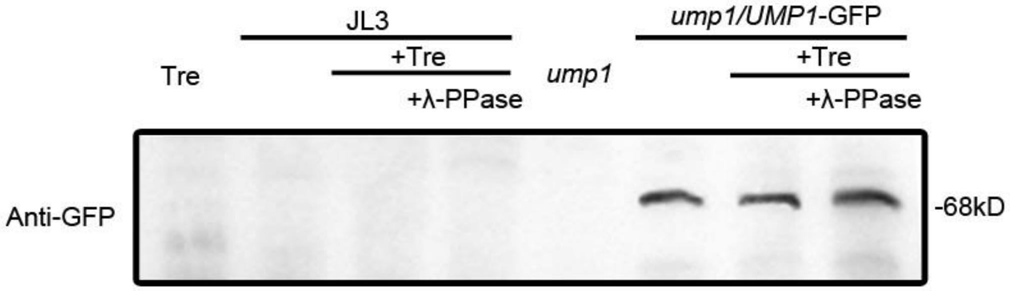

Supplementary Figure 7. Assays for the expression of Ump1-GFP fusion protein.

Western blots of proteins isolated from algal-fungal cocultures and algal or fungal cells were detected with anti-GFP antibody. The 68-kD Ump1-GFP fusion protein was detected in *ump1/UMP1*-GFP strains but not in JL3 or the *ump1* mutant. Expression of Ump1-GFP fusion protein was not reduced in cocultures of algal and *ump1/UMP1*-GFP strain.

Supplementary Table 1. Number of algal cells with FDA and chlorophyll fluorescence signal in primary lichen thalli and three-month-old algal samples.

	FDA (%)	Chlorophyll (%)	Survival rate [a] (% with both signals)
**Primary lichen thalli**	78.3 ± 1.7	96.0 ± 2.2	75.3 ± 2.0
**3-month-old algae**	8.0 ± 0.8	12.7 ±1.7	7.7 ± 0.9

[a] Percentage of algal cells with FDA staining, chlorophyll fluorescence, and both signals. Only cells with both FDA and chlorophyll fluorescence signals were considered to be photosynthetically active to estimate the survival rate. Mean and standard errors are estimated with data from three (*n=3*) replicates with at least 100 algal cells examined in each replicate.

Supplementary Table 2. Primers used in this study.

**Primer**	**Sequence (5’-3’)**	**Application**
**1F**	TGCGAGGGGATGTTAAGGGA	*UMP1* knockout
**2R**	AATGCTCCTTCAATATCATCTTCTGTACTTACTAACTCATGGCTGC	*UMP1* knockout
**3F**	CGTCCGCAATGTGTTATTAAGTCGACGTTGTAAGATTAGATGGTCG	*UMP1* knockout
**4R**	AGTATACTTTCCTGCGTCGG	*UMP1* knockout
**5F**	TACGGCTCCAGGTACGGGT	*UMP1* knockout
**6R**	ATCCAGGTATGCTAAACCTCA	*UMP1* knockout
**7F**	AGGATTTCCGGGTGGAGTT	*UMP1* knockout
**8R**	CCACGGTTGTGACAGTCGCT	*UMP1* knockout
**HT-F**	ACAGAAGATGATATTGAAGGAGC	*hph* cassette
**HT-R**	GTCGACTTAATAACACATTGCGGACGT	*hph* cassette
**HY-R**	GTATTGACCG ATTCCTTGCG GTCCGAA	*hph* cassette
**YG-F**	GATGTAGGAGGGCGTGGATATGTCCT	*hph* cassette
**H850**	TTCCTCCCTTTATTTCAGATTCAA	*hph* cassette
**H852**	ATGTTGGCGACCTCGTATTGG	*hph* cassette
**C1F**	AGGGAACAAAAGCTGGGTACCCCGACCTCCAAGCCCCTCACGT	*UMP1* complementation
**C2R**	TCGCCCTTGCTCACCATAAGCTTATAAAGATCGAAGCAAGAGGGA	*UMP1* complementation
**C3F**	ATGGACGAGCTGTACAAGTAAACTTACTAACTCATGGCTGCA	*UMP1* complementation
**C4R**	GATGATTTCAGTAACGTTAAGTATTTATCGAATGAAGACTTTC	*UMP1* complementation

Supplementary Table 3. Accession numbers and genome locations of MAPKs used in phylogenetic analysis.

Organism	Gene name	GenBank accession or Gene number
Saccharomyces cerevisiae	Kss1	NP_011554.3 (https://www.ncbi.nlm.nih.gov/protein/NP_011554.3/)
	Fus3	CAA84835.1 (https://www.ncbi.nlm.nih.gov/protein/CAA84835.1)
	Slt2	KZV10752.1 (https://www.ncbi.nlm.nih.gov/protein/KZV10752.1)
	Hog1	CAA97680.1 (https://www.ncbi.nlm.nih.gov/protein/CAA97680.1)
Magnaporthe oryzae	Pmk1	MGG_09565 (https://www.ncbi.nlm.nih.gov/protein/G4N0Z0.1)
	Mps1	MGG_04943 (https://www.ncbi.nlm.nih.gov/protein/G4N374.1)
	Osm1	MGG_01822 (https://www.ncbi.nlm.nih.gov/protein/Q9UV51.1)
Fusarium graminearum	Gpmk1	FGSG_06385 (https://www.ncbi.nlm.nih.gov/protein/XP_011325047.1)
	Mgv1	FGSG_10313 (https://www.ncbi.nlm.nih.gov/protein/XP_011319273.1)
	FgHog1	FGSG_09612 (https://www.ncbi.nlm.nih.gov/protein/P0C431.1)
Umbilicaria muhlenbergii	Ump1	contig_264:164455-165751 (https://www.ncbi.nlm.nih.gov/nuccore/JFDN01000264.1?report=fasta)
	Um05c2270	contig_264:279110-280704 (https://www.ncbi.nlm.nih.gov/nuccore/JFDN01000264.1?report=fasta)
	Um22c2410	contig_70:173179-174700 (https://www.ncbi.nlm.nih.gov/nuccore/JFDN01000070.1?report=fasta)
Cladonia grayi	CLAGR_000041	scaffold_00001:120973-122903 (https://mycocosm.jgi.doe.gov/cgi-bin/dispGeneModel?db=Clagr3&id=42)
	CLAGR_007554	scaffold_00063:127726-129855 (https://mycocosm.jgi.doe.gov/cgi-bin/dispGeneModel?db=Clagr3&id=7690)
	CLAGR_008240	scaffold_00082:99984-101689 (https://mycocosm.jgi.doe.gov/cgi-bin/dispGeneModel?db=Clagr3&id=8685)
Endocarpon pusillum	EPUS_05302	scaffold_583:10969-12183 (https://www.ncbi.nlm.nih.gov/protein/ERF71250.1)
	EPUS_05320	scaffold_583:75529-77021 (https://www.ncbi.nlm.nih.gov/protein/ERF71268.1)
	EPUS_05777	scaffold_1:11930-14783 (https://www.ncbi.nlm.nih.gov/protein/ERF77208.1)
Acarospora strigata	AST1_003929	scaffold_8982:7250-8680 (https://mycocosm.jgi.doe.gov/cgi-bin/dispGeneModel?db=Acastr1&id=127093)
	AST1_001046	scaffold_3156:181472-183073 (https://mycocosm.jgi.doe.gov/cgi-bin/dispGeneModel?db=Acastr1&id=122930)
	AST1_001902	scaffold_19909:16945-18531 (https://mycocosm.jgi.doe.gov/cgi-bin/dispGeneModel?db=Acastr1&id=121002)
