# Supplementary material for: Regulation of symbiotic interactions and primitive lichen differentiation by UMP1 MAP kinase in Umbilicaria muhlenbergii
Source: Nat Commun. 2023 Nov 1;14:6972. doi: 10.1038/s41467-023-42675-8 (PMC10620189; doi:10.1038/s41467-023-42675-8)
Supplement: Supplementary file 2 — Reporting Summary [file 41467_2023_42675_MOESM2_ESM.pdf]

nature portfolio

Corresponding author(s): Jin-Rong Xu

Last updated by author(s): Oct 8, 2023

Reporting Summary

Nature Portfolio wishes to improve the reproducibility of the work that we publish. This form provides structure for consistency and transparency in reporting. For further information on Nature Portfolio policies, see our Editorial Policies and the Editorial Policy Checklist.

Statistics

For all statistical analyses, confirm that the following items are present in the figure legend, table legend, main text, or Methods section.

n/a	Confirmed	
☐	☒	The exact sample size (*n*) for each experimental group/condition, given as a discrete number and unit of measurement
☐	☒	A statement on whether measurements were taken from distinct samples or whether the same sample was measured repeatedly
☐	☒	The statistical test(s) used AND whether they are one- or two-sided<br>*Only common tests should be described solely by name; describe more complex techniques in the Methods section.*
☐	☒	A description of all covariates tested
☐	☒	A description of any assumptions or corrections, such as tests of normality and adjustment for multiple comparisons
☐	☒	A full description of the statistical parameters including central tendency (e.g. means) or other basic estimates (e.g. regression coefficient) AND variation (e.g. standard deviation) or associated estimates of uncertainty (e.g. confidence intervals)
☐	☒	For null hypothesis testing, the test statistic (e.g. *F*, *t*, *r*) with confidence intervals, effect sizes, degrees of freedom and *P* value noted<br>*Give P values as exact values whenever suitable.*
☐	☒	For Bayesian analysis, information on the choice of priors and Markov chain Monte Carlo settings
☒	☐	For hierarchical and complex designs, identification of the appropriate level for tests and full reporting of outcomes
☒	☐	Estimates of effect sizes (e.g. Cohen's *d*, Pearson's *r*), indicating how they were calculated

Our web collection on statistics for biologists contains articles on many of the points above.

Software and code

Policy information about availability of computer code

Data collection	No software was used.
Data analysis	Phylogenetic analyses were conducted with the RAxML-HPC BlackBox with 1000 bootstrap tests at the CIPRES Science Gateway website (http://www.phylo.org). Amino acid sequences were aligned with ClustalW and visualized with T-coffee (http://tcoffee.crg.eu).

For manuscripts utilizing custom algorithms or software that are central to the research but not yet described in published literature, software must be made available to editors and reviewers. We strongly encourage code deposition in a community repository (e.g. GitHub). See the Nature Portfolio guidelines for submitting code & software for further information.

Data

Policy information about availability of data

All manuscripts must include a data availability statement. This statement should provide the following information, where applicable:

- Accession codes, unique identifiers, or web links for publicly available datasets
- A description of any restrictions on data availability
- For clinical datasets or third party data, please ensure that the statement adheres to our policy

GenBank data: NP_011554.3, CAA84835.1, KZV10752.1, CAA97680.1, G4N0Z0.1, G4N374.1, Q9UV51.1, XP_011325047.1, /XP_011319273.1, P0C431.1, JFDN01000264.1, JFDN01000070.1, ERF71250.1, ERF71268.1, ERF77208.1. and JGI data: CLAGR_000041, CLAGR_007554, CLAGR_008240, AST1_003929, AST1_001046, AST1_001902 were used in this study to construct phylogenetic tree. Data supporting the major findings of this work are available within the paper

and its Supplementary Information file. Source data are provided with this paper.

Research involving human participants, their data, or biological material

Policy information about studies with human participants or human data. See also policy information about sex, gender (identity/presentation), and sexual orientation and race, ethnicity and racism.

Reporting on sex and gender	not applicable (nothing to do with human data)
Reporting on race, ethnicity, or other socially relevant groupings	not applicable
Population characteristics	not applicable
Recruitment	not applicable
Ethics oversight	not applicable

Note that full information on the approval of the study protocol must also be provided in the manuscript.

Field-specific reporting

Please select the one below that is the best fit for your research. If you are not sure, read the appropriate sections before making your selection.

☒ Life sciences ☐ Behavioural & social sciences ☐ Ecological, evolutionary & environmental sciences

For a reference copy of the document with all sections, see nature.com/documents/nr-reporting-summary-flat.pdf

Life sciences study design

All studies must disclose on these points even when the disclosure is negative.

Sample size	Assays for symbiotic interactions on glass were carried out in five (n=5) independent experiments. For assays with cellulose membranes, three (n=3) independent experiments were conducted. For cryo-SEM examination, fifty fungal-algal complexes and three individual lichen thalli were observed in each replicate. Data from three (n=3) replicates with at least 100 algal cells examined in each replicate were used to estimate the algal survival rate. The average growth rate of the wild type and ump1 mutant was determined with data from three independent repeats (n=3). For TpEY assays, the relative phosphorylation level of Ump1 in each treatment was estimated with data from three independent experiments and analyzed with the two-tailed Student's t test. Localization of Ump1-GFP to the nucleus were performed with data from three independent biological replicates.
Data exclusions	No data were excluded.
Replication	All experiments include at least three replicates, the results were similar for all replications.
Randomization	Fungal and algal cells were examined randomly under a microscope.
Blinding	Blinding tests with fungal or algal cultures or fungal-algal co-cultures are impossible. They differ so much and it is obvious to tell fungal cultures from algal cultures or fungal-algal co-cultures.

Reporting for specific materials, systems and methods

We require information from authors about some types of materials, experimental systems and methods used in many studies. Here, indicate whether each material, system or method listed is relevant to your study. If you are not sure if a list item applies to your research, read the appropriate section before selecting a response.

Materials & experimental systems

n/a	Involved in the study
☐	☒ Antibodies
☒	☐ Eukaryotic cell lines
☒	☐ Palaeontology and archaeology
☒	☐ Animals and other organisms
☒	☐ Clinical data
☒	☐ Dual use research of concern
☒	☐ Plants

Methods

n/a	Involved in the study
☒	☐ ChIP-seq
☒	☐ Flow cytometry
☒	☐ MRI-based neuroimaging

Antibodies

Antibodies used

The phosphor-p44/42 MAPK antibody was purchased from Cell Signaling Technology (Danvers, USA), Cat. no. 4370, Dilution 1:1000.
The anti-GFP antibody was purchased from Beyotime (Shanghai, China), Cat. no. AF0159, Dilution 1:1000.
The anti-Gpmk1 antibody was generated at the ABclonal Biotechnology (Wuhan, China) by injecting rabbits with a synthetic peptide of Gpmk1 of Fusarium graminearum (332-347 aa; DFDKHKDNLSKEQLKQ) that is highly conserved in Ump1 (332-347 aa), Dilution 1:5000.

Validation

The primary antibody phosphor-p44/42 MAPK and anti-Gpmk1 used in this study have been published in earlier studies in Fusarium graminearum. Manufacturer's website states that the anti-GFP antibody validated in all species. These three antibodies were further validated with Umbilicaria muhlenbergii in this study.
